# Determination of Phenolic Acids in Sugarcane Vinasse by HPLC with Pulse Amperometry

**DOI:** 10.1155/2018/4869487

**Published:** 2018-01-21

**Authors:** P. V. Freitas, D. R. da Silva, M. A. Beluomini, J. L. da Silva, N. R. Stradiotto

**Affiliations:** ^1^Departamento de Tecnologia de Alimentos, Centro de Ciências Exatas e Tecnológicas, Universidade Federal de Viçosa, Av. Peter Henry Rolfs s/n, Viçosa, MG, Brazil; ^2^Departmento de Química Analítica, Instituto de Química (IQ/CAr), Universidade Estadual Paulista, R. Prof. Francisco Degni 55, Jardim Quitandinha, 14800-060 Araraquara, SP, Brazil; ^3^Universidade Federal Fluminense, Escola de Engenharia Industrial e Metalúrgica de Volta Redonda, Av. dos Trabalhadores 420, Vila Santa Cecília, Volta Redonda, RJ, Brazil

## Abstract

A reversed-phase liquid chromatographic separation with pulsed amperometric detection of phenolic acids at a glassy carbon electrode is described. Chromatographic separation was carried out in isocratic conditions using 0.20 mol·L^−1^ acetic acid (pH 5.0)/water (80 : 20, *v*/*v*) as mobile phase under constant working potential mode of 0.80 V. Chromatographic peaks presented high resolution and separation. Calibration curves exhibited excellent correlation coefficients, above 0.995. Linear ranges of the analytes, in mg L^−1^, were of 0.018–18 (gallic acid), 0.146–19 (vanillic acid), 0.13–17 (caffeic acid), 0.016–16 (ferulic acid), and 0.008–17 (p-coumaric acid), respectively. Limits of detection ranged from 1.6 to 97 *μ*g·L^−1^ and precision varied in 1.73–3.78% interval. Concentrations of 19 ± 0.51 mg·L^−1^ and 7.8 ± 2.5 mg·L^−1^ were found for vanillic and caffeic acids, respectively, in a sugarcane vinasse sample. Gallic, ferulic, and p-coumaric acids were not detected. Recovery results demonstrated that the proposed method is accurate, and it can be used to detect and quantify phenolic acids in sugarcane vinasse without any influence of interferents.

## 1. Introduction

Sugarcane vinasse is an acidic brownish liquid generated after fermentation and distillation of juice for production of ethanol [1, 2]. Usually, each liter of ethanol produced results in 10 to 13 liters of sugarcane vinasse [3]. About 93% of vinasse is water, and 75% of dry matter consists of organic compounds mainly glycerol, ethanol, lactic and acetic acids, and phenolics [1, 4–7].

This waste has been usually applied as fertilizer on sugarcane crops due to its high contents of phosphorous and potassium [8]. However, such activity might cause several changes in soil such as elevation of pH, salinization, and leaching of metals to underground waters [9, 10]. Anaerobic digestion of vinasse is an attractive alternative to treat this waste, considering pollutant control and energy generation at the end of the process [11, 12]. However, presence of phenolics might result in low gas production levels and instability, slowing the expansion of this technique around the world [11–15]. Siles et al. [13] and Jiménez et al. [16] determined a total concentration of polyphenolic compounds of 450 mg·L^−1^. García-García et al. [17] and Bhattacharyya et al. [18] detected even higher amounts of phenolics in sugarcane vinasse samples, reporting values of 469 and 667, both in mg L^−1^. These results show that this waste has a greater phenolics content than beverages such as beer (60–100 mg·L^−1^) and white wine (200–300 mg·L^−1^) according to Bravo [19].

Phenolics can be subdivided in phenols and polyphenols. Phenols are characterized by one or more hydroxyl groups bonded to a single aromatic ring. Polyphenols are constituted by multiple phenol rings connected to each other which are responsible for plant defense against ultraviolet radiation and also provide resistance to pathogens [19–21]. Main groups of polyphenols are phenolic acids, flavonoids, and tannins [20].

Several reports introduced new approaches to reduce concentration of these compounds, which are toxic to anaerobic microorganisms, responsible for conversion of vinasse into biogas [6, 13, 16, 17, 22]. Efficiency of such proposed method is usually evaluated by determination of “total phenolics,” a sum of phenols, and polyphenols concentrations, by employing the spectrophotometric method reported by Folin and Ciocalteu [23, 24]. This procedure is also very common in industries, especially in wine factories, because of its simplicity and low financial cost [25]. However, Folin–Ciocalteu's method exhibits positive deviations from actual phenolics concentration due to a set of interferents like ascorbic acid and sulphites, for instance, since every molecule susceptible to oxidation accounts for generation of the chromophore species [24, 26].

Polyphenols are characterized by their innate electroactivity and ability to donate electrons which are both related to their antioxidant properties [25]. Such features encouraged the application of electroanalytical techniques to determine polyphenols in several matrices. Glassy carbon electrode (GCE) is usually chosen as the working electrode for polyphenols detection by HPLC or voltammetric methods. Cyclic voltammetry and HPLC-DAD were employed to determine eight polyphenols in wine [27]. Better results were achieved by using such techniques in relation to Folin–Ciocalteu method. Three phenolic acids and three flavonoids in red wine were studied by differential pulse voltammetry, HPLC-UV, and Folin–Ciocalteu procedure [28]. Positive deviations were observed with the spectrophotometric method due to presence of ascorbate and sulphite in addition to reducing sugars. Regarding HPLC-ECD for determination of polyphenols, there are several reports that analyzed different matrices such as wine [29, 30], wort and beer [31, 32], brandy [33], olive oil [34], citrus honey [35], propolis [36], fruits and vegetables [37], and Guanxintong tablets [38].

However, a major drawback for detection of some organic compounds such as polyphenols by HPLC-ECD is their strong tendency of adsorption and even polymerization on the electrode surface. Pulsed amperometric detection (PAD) is an interesting alternative to solve this problem. According to LaCourse, Jackson, and Johnson, PAD exploits the catalytic activity of oxides located on the electrode surface by promoting its cleaning and reactivation and, thus, anodic detection of an electroactive species can be achieved [39]. Agüí et al. [40] employed PAD to determine phenolics with high molecular weight by different voltammetric techniques with a GCE. Those analytes and their oxidation products tended to be adsorbed on the electrode surface resulting in decrease of peak currents, and use of PAD promoted stability of the analytical signal. A HPLC-ECD method employing PAD at a GCE was used for the determination of polyphenolic compounds in artichoke extracts and olive mill wastewaters [41]. In that work, the authors verified the electrode fouling due to adsorption of by-products which resulted in loss of signal under constant potential (DC) electrochemical detection. This issue was not observed by PAD, and sensitivity remained constant over the whole chromatographic run, demonstrating the importance of a pulse sequence to clean and reactivate the GCE surface.

To the best of our knowledge, only one work considered the use of HPLC to study polyphenols in vinasse [42]. Eight phenolic acids in sugar beet vinasse were studied by HPLC-UV. However, this proposed method demanded two different wavelengths for detection of the studied analytes. At 280 nm, only syringic acid could be quantified (56 ± 8.0 ppm) while, at 320 nm, 4.00 ± 2.00 ppm of p-coumaric acid and 12 ± 3.00 ppm of ferulic acid were found. After a saponification step, 71 ± 9.0 ppm of ferulic acid was found since this compound could be present as ferulates. Considering the lack of studies employing a sensitive, selective, fast, and easily operated procedure to determine polyphenols in sugarcane vinasse, this work developed a method for detection and quantification of phenolic acids in a vinasse sample by HPLC-ECD with PAD employing a GCE.

## 2. Experimental

### 2.1. Reagents and Solutions

Standard solutions of caffeic, p-coumaric, gallic, and vanillic acids were prepared with concentration of 901, 821, 850, and 841 mg·L^−1^, respectively, by using analytical grade chemicals from Sigma-Aldrich. Standard solution of ferulic acid was prepared with a concentration of 971 mg·L^−1^ from a reagent (>98%) obtained from Fluka Chemie. Boiling ultrapure water (MILLI-Q) was employed for dissolution of the studied phenolic acids due to low solubility of the analytes in water at 25C.

### 2.2. Apparatus

Cyclic voltammograms were obtained by means of an Autolab PGSTAT 30 potentiostat controlled by NOVA 1.11 software. The working electrode was a GCE (3.00 mm diameter). Ag/AgCl (KCl 3.0 mol·L^−1^) and a Pt wire were used as reference electrode and counter electrode, respectively. Several buffer solutions—acetate buffer (pH 3.60 and 5.0), phosphate buffer (pH 7.0 and 8.9), and NaOH (pH 13)—were evaluated as supporting electrolytes in order to determine the best conditions for chromatographic separation of the studied analytes. Potential ranged from −0.15 V to 1.35 V, and scan rate was of 50 mVs^−1^.

Separation of phenolic acids was carried out by an ion chromatograph 850 Professional IC Cation-HP-Gradient (Metrohm) with extender module (pump C, postcolumn), injection loop of 20 *μ*L, and 863 compact autosampler (Metrohm). Pulsed amperometric measurements in flowing streams were performed using a Model IC amperometric detector (Metrohm) and an electrochemical wall-jet cell (Metrohm) consisting of a GCE as working electrode (3.00 mm diameter), a palladium (Pd) reference electrode, and a platinum (Pt) counter electrode. A computer equipped with a Magic Net, version 3.1, software enabled the acquisition and processing of the chromatograms. Chromatographic separation of polyphenols was performed by using a Hypersil GOLD C8 (Thermo Scientific) column (150 × 3.00 mm, 5.0 *μ*m) and a Hypersil GOLD C8 (Thermo Scientific) guard column (10 × 3.00 mm). Temperature of column furnace and detector was of 35°C. Mobile phase composition and flow rate were optimized, as described later in Section 3.3, and consisted of 0.20 mol·L^−1^ acetate buffer (pH 5.0)/ultrapure water (80 : 20 *v*/*v*) at 0.8 mL·min^−1^, isocratic elution. PAD of phenolic acids consisted of a three pulse-sequence. In the first pulse (E1), detection of analytes was performed. Due to adsorption and polymerization of molecules generated by E1, a second potential (E2), which is more positive than the previous pulse, was applied in order to oxidize all compounds present on electrode surface. Finally, a negative third potential (E3) was sought to promote reduction of species that could remain adhered to the GCE [39]. Electrochemical detection was performed by an optimized pulse sequence (Section 3.3): E1, 0.80 V for 300 ms; E2, 1.10 V for 50 ms; and E3, −0.10 V for 200 ms.

### 2.3. Sample Preparation

Sugarcane vinasse sample was obtained from a sugar and ethanol plant located near Araraquara, Brazil. The sample was centrifuged at 4000 rpm for 20 min in order to remove mineral impurities present in the sample. Then, the supernatant was collected and filtered (Millipore filters 5.0, 0.47, and 0.22 *μ*m). Samples were injected in HPLC with a dilution factor of 1 : 10 and 9 : 10, both in *v* : *v*, in ultrapure water.

## 3. Results and Discussion

### 3.1. Effect of pH on Electrochemical Oxidation of Analytes

**Figure 1:**
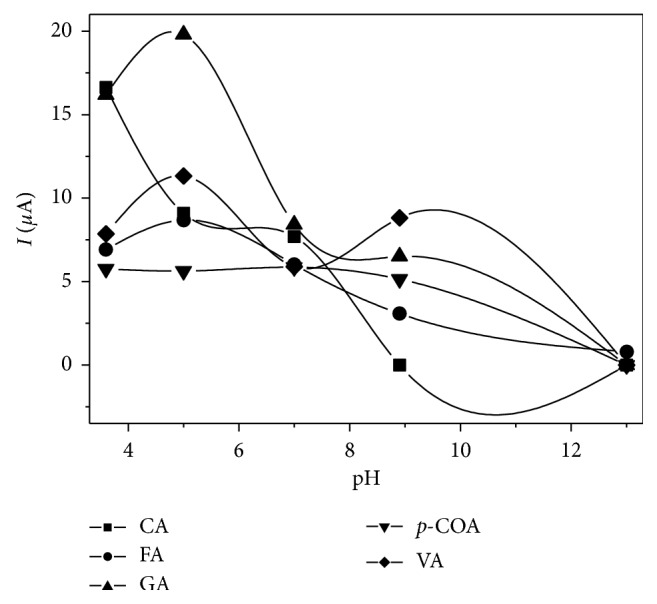
Effect of pH on first anodic peak current of studied phenolic acids employing 0.1 mol·L^−1^ buffers and scan rate of 50 mVs^−1^. CA: caffeic acid (180.2 mg·L^−1^); FA: ferulic acid (194.2 mg·L^−1^); GA: gallic acid (170.1 mg·L^−1^); p-COA: p-coumaric acid (164.2 mg·L^−1^); VA: vanillic acid (168.2 mg·L^−1^).

Influence of pH on first anodic peak currents (*I*_pa_) related to oxidation of tested polyphenols was evaluated by using cyclic voltammetric experiments employing different 0.1 mol L^−1^ solutions. Acetate buffer (pH 3.60 and 5.0), phosphate buffer (pH 7.0 and 8.9), and NaOH (pH 13) solutions were used. Potentials ranged from −0.10 to 1.35 V, and a scan rate of 50 mV s^−1^ was employed. Cleaning of the working electrode was performed after every measurement.  Figure 1 exhibits *I*_pa_ values observed for each analyte at these conditions. Acetate buffer solution (pH 3.60) gave the highest *I*_pa_ for caffeic acid while pH 5.0 acetate buffer presented similar effects for ferulic, gallic, and vanillic acid. Regarding p-coumaric acid, great variations were not observed in pH values of 3.60, 5.0, and 7.0. Use of basic solutions (pH 8.9 and pH 13) resulted in a decrease of *I*_pa_ values. Only for ferulic acid, an anodic peak was observed in NaOH solution. Those results can be explained by analysis of pK_*a*_ values of studied polyphenols, which range from 4.50 to 9.5, making them weak acids [43]. Thus, acetate buffer (pH 5.0) was chosen for subsequent experiments since all phenolic acids exhibited relatively high signals in this supporting electrolyte.

### 3.2. Cyclic Voltammetry

**Figure 2:**
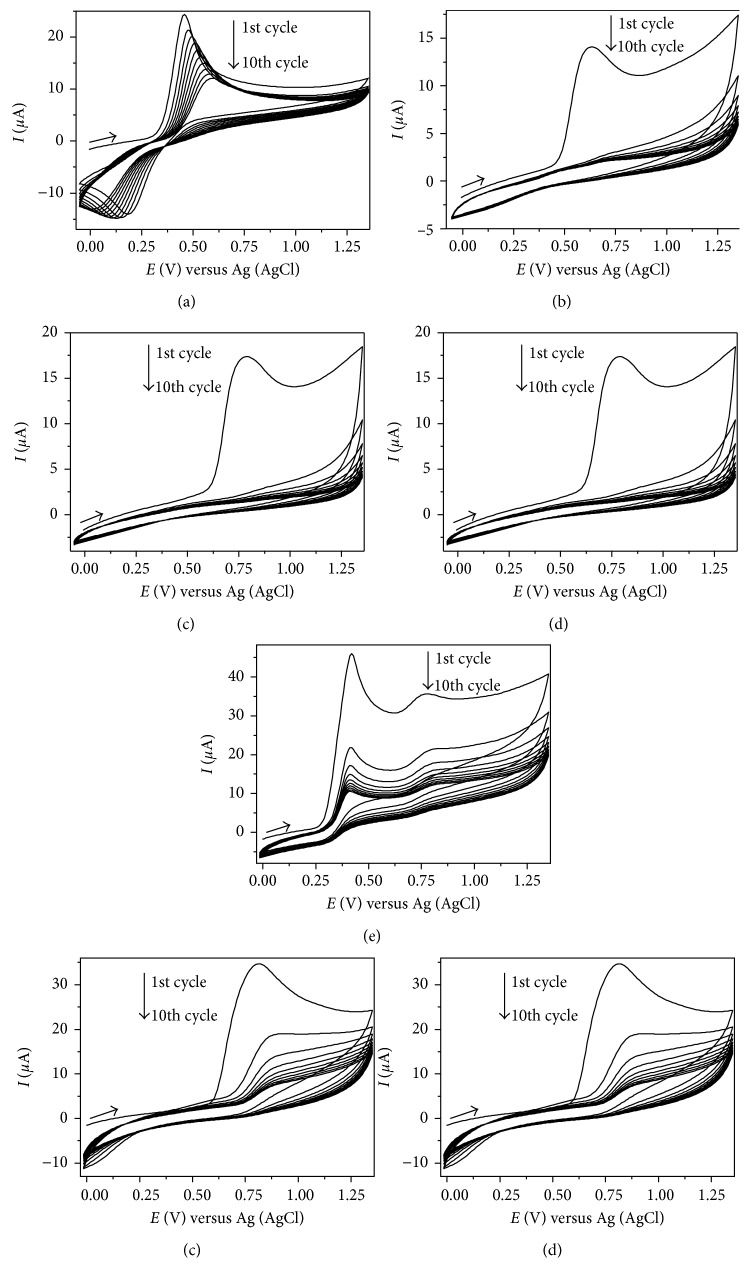
Cyclic voltammograms of studied polyphenols in 0.1 mol·L^−1^ acetate buffer (pH 5.0) and potential range of −0.15 to 1.35 V. (a) Caffeic acid (180.2 mg L^−1^); (b) ferulic acid (194.2 mg·L^−1^); (c) p-coumaric acid (164.2 mg·L^−1^); (d) gallic acid (170.1 mg·L^−1^); (e) vanillic acid (168.2 mg·L^−1^). Scan rate: 50 mV·s^−1^.

Figure 2 presents the cyclic voltammograms obtained for each studied phenolic acid after ten successive scans in a potential range of −0.15 to 1.35 V. These experiments were conducted without cleaning the GCE between each cycle in order to verify whether adsorption of generated products occurred or not. Such phenomenon would result in a decrease of measured currents as the scans were repeated due to fouling of the electrode surface. Anodic peak potentials (*E*_pa_) of caffeic and vanillic acids were detected in 0.40–0.60 V and 0.80–0.90 V potential intervals, respectively. Anodic peaks observed for ferulic and p-coumaric acids were around 0.60 V and 0.80 V, respectively. Gallic acid exhibited a different pattern from all analytes with two anodic peaks. The first peak was more intense and emerged at 0.40 V. According to Brenna et al. [29], o- or p-diphenols present *E*_pa_ values that range from 0.40 to 0.60 V (versus Ag/AgCl) due to their electrochemical oxidation to o- or p-quinones. When a hydroxyl group or two –OH groups in meta-position are present, oxidation only occurs at higher potentials (0.80 to 1.10 V) while molecules with three hydroxyl groups exhibit an anodic peak around 0.45 V.

**Figure 3:**
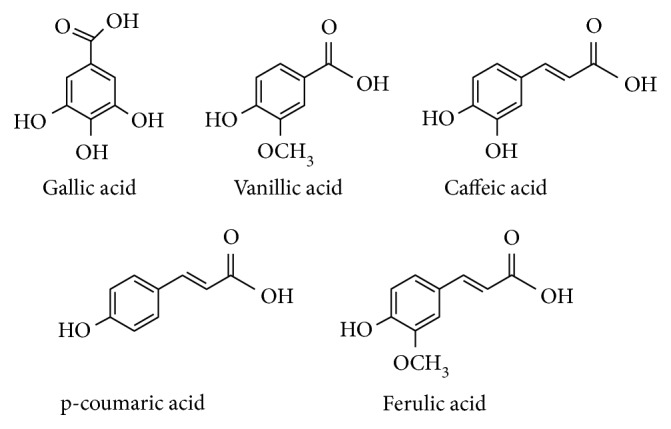
Molecular structures of studied phenolic acids.

Figure 3 shows the molecular structure of the studied phenolic acids. For ferulic acid, oxidation reaction involved loss of a hydrogen atom from the hydroxyl group bonded to the aromatic ring. A phenoxyl radical was generated which is highly stabilized by resonance, since the lone electron is dislocated over the whole molecular structure and not on oxygen atom only. Thus, the phenoxyl radical presents low reactivity and is not able to start or propagate reactions of the oxidative chain, finishing such process by reaction with another phenoxyl radical and yielding phenolic products, which also present antioxidant activity [42–44]. Single anodic peaks of caffeic and p-coumaric acids and first anodic peak of gallic acid are related to oxidation of hydroxyl groups located in aromatic ring of those phenolic acids. Vanillic acid presents in its molecular structure an ether functional group (–OCH_3_) bonded to the aromatic ring. Such group, in addition to the hydroxyl group attached to the next carbon atom in benzene ring, is oxidized in a two electron transfer process [27].

It can be seen from Figure 2 that measured currents were lowered as voltammetric cycles were repeated due to adsorption of oxidation products on GCE surface. Makhotkina and Kilmartin [27] reported similar results and verified that the saturation surface coverage (*Γ*_*s*_) of the electrode ranged from 0.20 to 1.00 nmol·cm^−2^. Considering phenolic acids, greater values of this parameter were estimated for hydroxycinnamic acids in relation to benzoic acids [27]. Caffeic, ferulic, and p-coumaric acids exhibited *Γ*_*s*_ of 0.70, 0.90, and 1.00 nmol·cm^−2^, respectively. For vanillic and gallic acids, lower values of *Γ*_*s*_ were observed (0.40 and 0.50 nmol·cm^−2^, resp.). Adsorption of caffeic acid on GCE by cyclic voltammetry was also reported by Silva et al. [45]. However, since vanillic, p-coumaric, and ferulic acids showed zero *I*_pa_ values in subsequent scans, it is possible that other processes such as polymerization have occurred. According to Hapiot et al. [46], the electrochemical oxidation of p-coumaric and ferulic acids involves an electron transfer from the corresponding phenolate ion. Such ion is oxidized to a phenoxyl radical which dimerizes by a radical-radical coupling.

### 3.3. Development of Chromatographic Method and Determination of Polyphenols in Sugarcane Vinasse Sample

Elution strength of the mobile phase was studied by using 0.20 mol·L^−1^ acetate buffer solution (pH 5.0) and water in isocratic mode. Concentration of acetate buffer in mobile phase was altered in order to avoid overlapping of detected peaks observed for every analyte. Also, that procedure was done to evaluate retention times of the studied polyphenols. Sequence of elution of the analytes in these conditions, from lower to higher retention times, was as follows: gallic, vanillic, caffeic, ferulic, and p-coumaric acids.

**Figure 4:**
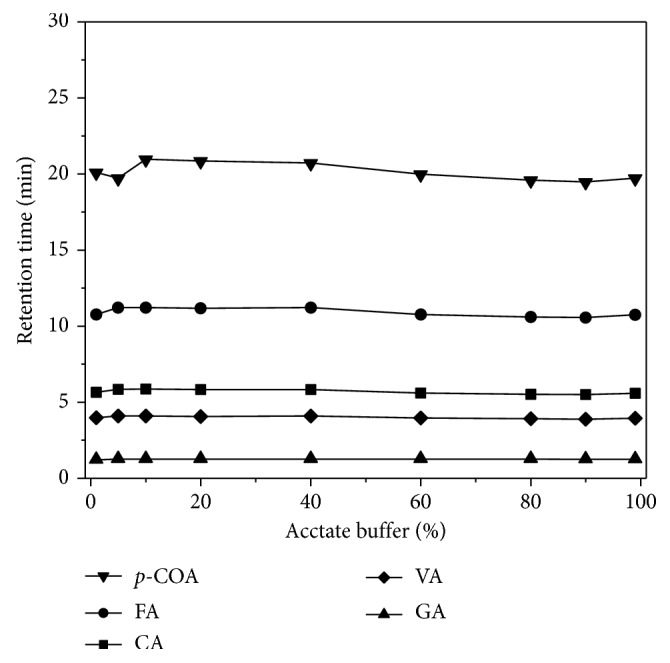
Effect of 0.20 mol·L^−1^ acetate buffer (pH 5.0) concentration on the retention times of a mixture of polyphenols. CA: caffeic acid (3.60 mg·L^−1^); FA: ferulic acid (3.88 mg·L^−1^); GA: gallic acid (3.40 mg·L^−1^); p-COA: p-coumaric acid (3.28 mg·L^−1^); VA: vanillic acid (3.36 mg·L^−1^). Conditions: isocratic elution with 0.20 mol·L^−1^ acetate buffer : ultrapure water as mobile phase; flow rate of 0.8 mL·min^−1^; detection potential of 0.80 V (versus Pd).

Considering caffeic, vanillic, and gallic acids, no significant changes were observed in retention times of these polyphenols with increment of acetate buffer concentration in mobile phase (Figure 4). Elevation of *t*_*r*_ values was verified for ferulic acid as proportion of acetate buffer increased until 40%. Then, such trend was inverted and, at 60%, retention time stabilized regardless of ratio of acetate buffer in mobile phase. A similar behaviour was observed for p-coumaric acid. However, maximum retention time occurred in 10% of acetate buffer in mobile phase followed by a decrease which was more intense after 40%. This tendency came to a halt when concentration of acetate buffer in mobile phase was of 90%. Then, when only acetate buffer was present in the mobile phase, increase of *t*_*r*_ values was observed. Based on *t*_*r*_ values of each phenolic acid observed in several proportions of 0.20 mol·L^−1^ acetate buffer (pH 5.0)/water mixture, 80 : 20 (*v*/*v*) proportion was chosen for subsequent experiments.

Retention of a compound in stationary phase is stronger when the solubility of such analyte in mobile phase is lower. Thus, sequence of elution of polyphenols is related to differences in their molecular structures, specially type, position, and number of functional groups attached to the aromatic ring. Presence of hydroxyl groups enhanced the mobility of a molecule and thus reduced retention time while compounds with methoxyl (-OCH_3_) groups exhibited higher retention times due to their lower mobility [37]. Gallic acid, which contains three hydroxyl and one carboxyl groups (Figure 3), was the analyte with highest polarity and, consequently, exhibited the lowest retention time. A larger carbonic chain is seen for C_3_H_3_O_2_ group when compared to OCH_3_ group. Such fact promoted the affinity of p-coumaric acid with the stationary phase and affected solubility of this compound in mobile phase. Thus, the highest retention times were detected for this analyte in relation to other studied compounds.

Other chromatographic conditions such as mobile phase flow and applied potential for polyphenols detection were studied. Increment of mobile phase flow rate resulted in decrease of retention times observed for each studied polyphenol. When mobile phase flow rate was higher than 1.00 mL·min^−1^, gallic acid was detected with very low retention times, undesirable for the analysis since the peak related to this phenolic acid was very close to the one belonging to unretained mobile phase. Considering that and the fact that the experiment is expected to be as fast as possible, a 0.80 mL·min^−1^ mobile phase flow rate was chosen for the subsequent experiments.

Since all analytes exhibited a heavy tendency to be adsorbed or even polymerize on GCE surface (Section 3.2) resulting in blocking and fouling of the sensor, a pulse sequence was established in order to clean this surface and to allow other molecules to reach the electrode. The sequence consisted of a detection potential (*E*1), a higher potential for complete oxidation of adsorbed species generated after application of *E*1 (*E*2 = 1.10 V), and a lower potential (*E*3 = −0.10 V) for reduction of remaining species that could be still adsorbed on the electrode and, thus, renewing its surface before a new pulse sequence.

**Figure 5:**
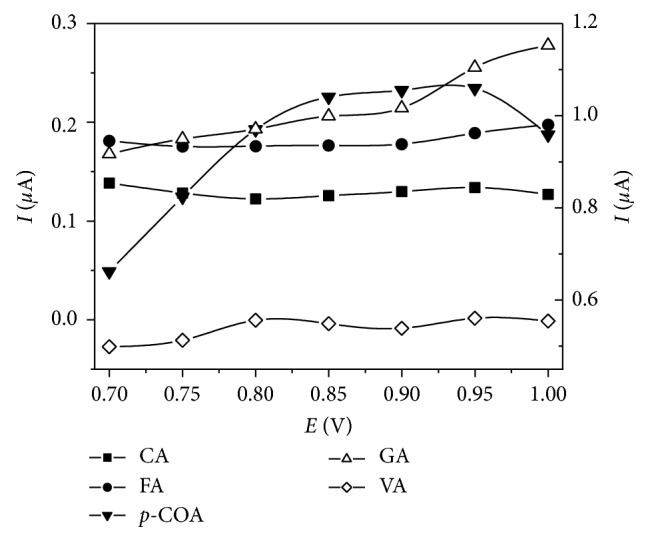
Plot of applied potential versus peak current for all analytes. Left axis (black symbols): CA: caffeic acid (3.60 mg·L^−1^); FA: ferulic acid (3.88 mg·L^−1^); p-COA: p-coumaric acid (3.28 mg·L^−1^). Right axis (white symbols): GA: gallic acid (3.40 mg·L^−1^); VA: vanillic acid (3.36 mg·L^−1^). Mobile phase: 0.20 mol·L^−1^ acetate buffer (pH 5.0)/ultrapure water (80 : 20 *v*/*v*). Other experimental conditions are as shown in Figure 4.

Variation of potential employed for detection of analytes (*E*1) did not result in significant changes of measured peak currents of caffeic, vanillic, and ferulic acids (Figure 5). Detection potential effect on anodic peak currents was evaluated in the 0.70 V–1.00 V interval. Despite gallic, ferulic, and caffeic acids presenting *E*_pa_ inferior than 0.70 V, potentials smaller than this value were not studied since vanillic and p-coumaric acids exhibited very low currents in such range, as it can be seen from their cyclic voltammograms in Figure 2. Since a simultaneous detection of the analytes is desirable, detection potential should be a value in which the analytes present the highest measured current considering the behaviour of all of them.

Gallic acid displayed increased peak currents in higher potentials which denoted a progressive elevation of detectability of this analyte. The highest sensitivity to applied potential variations was presented by p-coumaric acid. From 0.80 V to 0.95 V, peak areas presented maximum values while, in potentials greater than 0.95 V, a decrease of peak currents was detected. Taking into account the results obtained for every phenolic acid, potential of 0.80 V was applied in all subsequent experiments.

**Figure 6:**
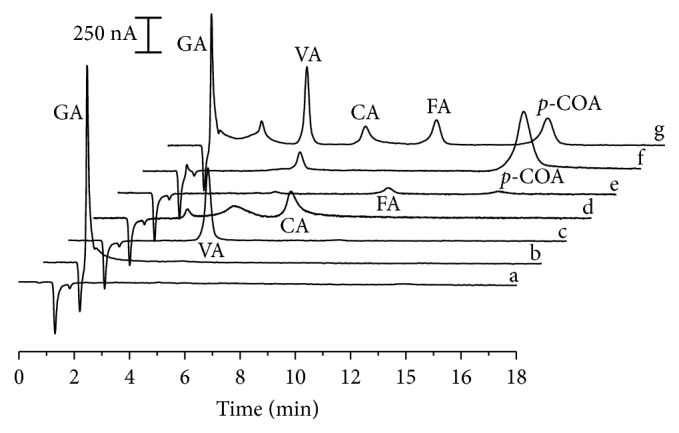
Chromatograms acquired for blank solution (a), gallic (b), vanillic (c), caffeic (d), ferulic (e), and p-coumaric acids (f) and for the mixture of standard solutions of the analytes (g). Potential: 0.80 V (versus Pd). Mobile phase: 0.20 mol·L^−1^ acetate buffer (pH 5.0)/ultrapure water (80 : 20 *v*/*v*). Isocratic elution. Mobile phase flow rate: 0.8 mL·min^−1^. CA: caffeic acid (3.60 mg·L^−1^); FA: ferulic acid (3.88 mg·L^−1^); GA: gallic acid (3.40 mg·L^−1^); p-COA: p-coumaric acid (3.28 mg·L^−1^); VA: vanillic acid (3.36 mg·L^−1^).

Thus, optimized condition for separation of studied phenolic acids is as follows: isocratic elution using a mixture of 0.20 mol·L^−1^ acetate buffer (pH 5.0) and ultrapure water (80 : 20, in %) and mobile phase flow of 0.80 mL·min^1^. By using such condition, Figure 6 displays the chromatograms acquired for each studied polyphenol and for the mixture containing standard solutions of these analytes as well as the blank. Despite not employing an organic solvent for their elution, all analytes were detected in almost fourteen minutes. Usually acetonitrile or methanol is added to the mobile phase for chromatographic separation of polyphenols on a gradient elution [33, 42, 47, 48] with a C18 column. However, the C8 chromatographic column employed in this work is less hydrophobic than a C18 one and, thus, polyphenols are less retained by the stationary phase in this condition. Furthermore, all of studied analytes at pH 5.0 are in the deprotonated form since they are weak acids. pK_*a*_ values for the analytes are as follows [49]: gallic acid: pK_*a*_ = 4.11; vanillic acid: pK_*a*_ = 4.17; caffeic acid: pK_*a*_ = 4.30; ferulic acid: pK_*a*_ = 4.30; p-coumaric acid: pK_*a*_ = 4.34. Consequently, they have a higher affinity by an aqueous mobile phase, and retention times of these species are lower in relation to their protonated forms in the same conditions. Also, concerning HPLC-ECD, employment of an organic solvent in mobile phase induced noise during chromatographic separation with PAD of furanic aldehydes as previously observed [50].

The chromatographic parameters such as dead time (*t*_*m*_), retention time (*t*_*r*_), adjusted retention time (*t*'_*r*_), selectivity factor (*α*), and capacity factor (*k*) were all found to be within the expected results for a good chromatographic performance as it can be seen in Table 1.

Only capacity factor calculated for gallic acid was located in a problematic region, lower than 1, denoting that the elution of this analyte occurred too fast. Nevertheless, such phenolic acid could be easily detected in chromatograms. Calibration curves were obtained for each studied analyte in order to assess analytical parameters such as limits of detection, amperometric sensitivity, and linear range. Concentration ranges employed were of caffeic acid (18 *μ*g·L^−1^–18 mg·L^−1^), ferulic acid (19 *μ*g·L^−1^–19 mg·L^−1^), gallic acid (17 *μ*g·L^−1^–17 mg·L^−1^), p-coumaric acid (16 *μ*g·L^−1^–16 mg·L^−1^), and vanillic acid (17 *μ*g·L^−1^–17 mg·L^−1^). Figures of merit for determination of polyphenols by HPLC-PAD are demonstrated in Table 2. Limits of detection (LOD) were defined as three times signal-to-noise ratio (*S*/*N*). LODs ranged from 1.6 to 97 *μ*g·L^−1^. In comparison with other reports that employed HPLC-ECD [35, 47, 48] for phenolic acid detection, LOD observed in this work for p-coumaric acid was lower. Such trend was also verified when CoulArray was employed for polyphenols detection [47, 51] with LODs estimated in the present study for vanillic and caffeic acids being similar or lower than previously reported. Use of PAD for cleaning and reactivation of GCE surface probably promoted such increase of sensitivity in relation to other methods, mainly when phenolic acids with high predisposition to adsorption and polymerization, as vanillic and p-coumaric acids, on the electrode surface. In addition, precision ranged in 1.73–3.78% interval.

**Figure 7:**
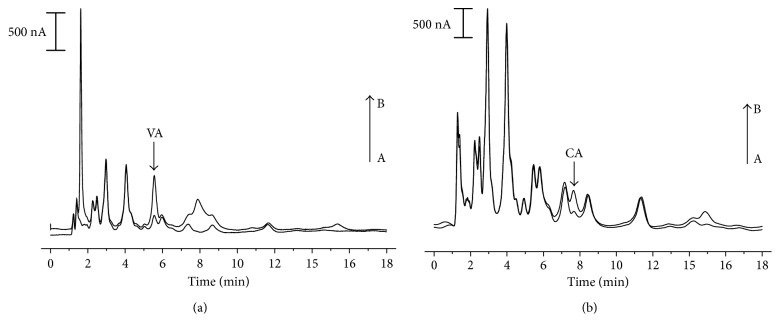
Chromatograms acquired for (a) sugarcane vinasse sample (1 : 10 dilution) (A) and sample with standard addition of 25 *μ*mol·L^−1^ (A). (b) Sugarcane vinasse sample (9 : 10 dilution) (A) and sample with standard addition of 50 *μ*mol·L^−1^ (B). Experimental conditions are as shown in Figure 4.

Polyphenols present in sugarcane vinasse sample were identified by their specific retention times and confirmed by the standard addition method (Figure 7). Four different concentrations of the studied phenolic acids were added to the sample: 7.5, 10, 25, and 50 *μ*mol·L^−1^. Figure 7 displays the obtained chromatogram with a 1 : 10 dilution ratio while Figure 7 shows the chromatogram of the same sample in a 9 : 10 dilution factor.

Gallic, p-coumaric, and ferulic acids could not be detected in the sample. Vanbeneden et al. [32], when determining hydroxycinnamic acids and volatile phenols in wort and beer by HPLC-ECD, verified that, for some samples, p-coumaric acid could not be quantified. According to the authors, probably the presence of a metabolite generated in the fermentation stage interfered in determination of this analyte.

Linear equations obtained by the standard addition method for vanillic acid and caffeic acid were, respectively, of *y* = 4.13·10^−6^*x* + 52.3 (*r* = 0.994) and *y* = 3.79·10^−6^*x* + 16.5 (*r* = 0.958). Method validation is displayed in Table 3. It was observed that vanillic acid was present in the highest concentration in sugarcane vinasse sample (19 ± 0.51 mg·L^−1^) followed by caffeic acid (7.8 ± 2.47 mg·L^−1^). Recovery of vanillic and caffeic acids was determined by standard addition method, and results indicated that the proposed method was accurate with relative standard deviation (RSD, in %) of 2.2 and 3.2, respectively.

## 4. Conclusions

Liquid chromatography with PAD at GCE was employed for the determination of phenolic acids using a reversed-phase column, isocratic elution, mobile phase composed of 0.20 mol·L^−1^ acetate buffer (pH 5.0) : ultrapure water (80 : 20, in %) with flow rate of 0.80 mL·min^−1^. This class of polyphenols could be determined in a sugarcane vinasse sample without any extraction or derivatization steps and chromatographic separation was accomplished after 18 minutes. Adsorption of phenolic compounds as well as their oxidation products on GCE surface was avoided by the use of PAD. When compared to other reported procedures, the proposed method described in this work could be an interesting alternative since it is fast, accurate, and able to detect and quantify phenolic acids, even in a complex sample such as sugarcane vinasse.

## Figures and Tables

**Table 1:** Chromatographic parameters for separation of polyphenols^a^.

Parameter	GA	VA	CA	FA	p-COA
*t*_*m*_ (min)	1.30	1.30	1.30	1.30	1.30
*t*_*r*_ (min)	1.58	5.00	7.10	9.70	13.7
*t'*_*r*_ (min)	0.28	3.72	5.80	8.40	12.4
*k*	0.22	2.86	4.49	6.50	9.60
*α*	1.00	13.3	20.9	30.0	44.4

^a^Experimental conditions are as shown in Figure 4; GA, gallic acid; VA, vanillic acid; CA, caffeic acid; FA, ferulic acid; p-COA, p-coumaric acid.

**Table 2:** Figures of merit for determination of polyphenols by HPLC-PAD.

Figure of merit	GA	VA	CA	FA	p-COA
Concentration range (mg L^−1^)	0.018–18.0	0.15–19.4	0.13–17.0	0.016–16.4	0.008–16.8
Amperometric sensibility (A L mg^−1^)	84 ± 1.89	49 ± 0.69	28 0.39	6.0 ± 0.14	56 ± 0.70
Correlation coefficient	0.996	0.997	0.998	0.995	0.998
LOD (mg L^−1^)	0.085	0.0042	0.0045	0.097	0.0016
Precision (%)	3.78	2.19	2.75	1.73	3.05

GA, gallic acid; VA, vanillic acid; CA, caffeic acid; FA, ferulic acid; p-COA, p-coumaric acid.

**Table 3:** Method validation.

Parameter	VA	CA
Original amount (mg L^−1^)	19 ± 0.51	7.8 ± 2.47
Added amount (mg L^−1^)	3.36	3.60
Recovery (%)	102	102
RSD (*n* = 3) (%)	2.19	2.75
